# Evaluation of the Rigo classification system for brace design

**DOI:** 10.1186/1748-7161-9-S1-O31

**Published:** 2014-12-04

**Authors:** Dimitris Papadopoulos

**Affiliations:** 1 Spondylos Laser Spine Lab, Athens, Greece

## Background

We were using for the design of scoliosis braces, the King classification system combined with the Cheneau brace concepts as well as the Lehnert’s 3-4 curves pattern. This was very frustrating either with the correction results or the body balance. We were completely confused and the design of a brace was empirical, based on the expert’s skill.

## Aim

The aim was to evaluate the Rigo classification system for the brace design.

## Design

From July 2011 we have started to fabricate the RSB braces for 243 exclusively AIS patients (220 females and 23 males, average age 13.3, with thoracic Cobb angle average 35 degrees and lumbar 29 degrees), based on the Rigo Classification system. The therapy was completed by Schroth and SEAS exercises and the suggested wearing time was 21 hours per day.

## Methods

The inter-observer reliability of the classification was testing, in every case individually by an orthopaedic surgeon with CPO degree and a Physiotherapist (Schroth and SEAS certified). The total of the cases for the purpose of this paper was checked again by the same team. The patients were visited every 3 months, checking posture and Cobb angle with Formetric 4D, as well the adequacy of the brace. The patients have had also an in brace x-Ray, about three months after bracing.

## Results

From the 243 patients we had 13 patients A1, 9 A2, 41 A3, 66 B1, 28 B2, 20 C1, 26 C2, 22 E1 and 18 E2, according the Rigo classification. The in brace x-rays have shown a correction from 2/3 to 1/3 of the initial Cobb angle, depending on the skeletal maturity, as well as the compliance of the patient. All the patients out of the brace had a better balance and aesthetics (Scapular symmetry, waist triangles, humps and pelvic displacement), almost from the first visit after 3 months.

## Conclusion

The Rigo classification system was verified to be excellent for RSB brace, which gave us the best results ever taken. We don’t know if it will be the same excellent for other brace types, such as Boston, Sforzesco etc.
